# Validation of the Proposed Specifiers for Conduct Disorder (PSCD) Scale in a Sample of Italian Students

**DOI:** 10.3390/children8111020

**Published:** 2021-11-06

**Authors:** Pietro Muratori, Carlo Buonanno, Anna Gallani, Giuseppe Grossi, Valentina Levantini, Annarita Milone, Simone Pisano, Randall T. Salekin, Gianluca Sesso, Gabriele Masi, Annalaura Nocentini

**Affiliations:** 1 IRCCS Stella Maris, Scientific Institute of Child Neurology and Psychiatry, 56128 Pisa, Italy; pietro.muratori@fsm.unipi.it (P.M.); valentina.levantini@hotmail.it (V.L.); annarita.milone@fsm.unipi.it (A.M.); gianluca.sesso@fsm.unipi.it (G.S.); 2 Scuola Psicoterapia Cognitiva, 00185 Roma, Italy; buonanno@apc.it (C.B.); g.grossi@hotmail.it (G.G.); 3 Specialized Centre for Learning Disabilities, Uonpia Ausl, 40127 Ferrara, Italy; anna.gallani@gmail.com; 4 Department of Translational Medical Sciences, Federico II University, 80138 Naples, Italy; pisano.simone@gmail.com; 5 Department of Psychology, The University of Alabama, P.O. Box 870348, Tuscaloosa, AL 35487, USA; rsalekin@ua.edu; 6 Department of Sciences of Education and Psychology, University of Florence, 50121 Firenze, Italy; annalaura.nocentini@unifi.it

**Keywords:** grandiose-manipulative, callous-unemotional, daring-impulsive, psychopathy, early adolescent, conduct problems, hyperactivity

## Abstract

This study aimed to further validate the self-reported version of the Proposed Specifiers Conduct Disorder (PSCD) scale, testing the associations between the PSCD with a scale that measures emotional/behavioral difficulties and prosocial behaviors (Strength and Difficulties Questionnaire, SDQ). A total of 536 Italian students (47.76% male; 11–14 years) completed the PSCD, while their caregivers and teachers completed the SDQ. A series of confirmatory factor analyses to test the best fitting model were run. The internal consistency of the PSCD was evaluated, and the correlations between the PSCD self-reported scores and SDQ Parent and Teacher report scores were examined. A bi-factor model was fitted with a refined 19-item version of the scale, which showed adequate fit indices. The PSCD total score was strongly associated with higher parent- and teacher-rated conduct problems, hyperactivity, and lower prosocial behavioral symptoms. In conclusion, this study indicated that the self-report PSCD shows preliminary promise as a reliable, easy-to-use tool, for measuring psychopathic traits in Italian children and young adolescents.

## 1. Introduction

Hare’s Psychopathy Checklist (PCL-R; [1]), in its revised version, describes psychopathy as a multidimensional construct encompassing at least four components: Interpersonal (i.e., superficial charm, grandiose self, manipulative tendencies); Affective (i.e., lack of guilt and remorse, shallow affects); Lifestyle/Behavioral (i.e., parasitism, sensation seeking, impulsivity); and Antisocial (i.e., delinquency, criminality). This general multidimensional structure has been replicated in factor analysis-based studies in adults [2,3], and similar findings have been confirmed in children and adolescents [4,5].

Recent research has increasingly focused on one dimension of psychopathy in children and adolescent populations, namely Callous Unemotional traits (CU). Such a trend likely finds its roots in the several studies showing that the levels of CU traits (lack of guilt, shallow emotions, and proactive aggression) are associated with a more persistent and severe pattern of aggressive behavior and delinquency and a greater risk for adverse outcomes throughout the lifespan (e.g., antisocial behavior, substance use, violent crimes, and detentions; [6,7,8,9,10]).

Nevertheless, some recent evidence suggests that the combination of all dimensions of psychopathy is more strongly related to and predictive of conduct problems in childhood than CU traits alone, and childhood psychopathy would be preferably represented by all its dimensions (see [11,12]). Therefore, some tools have been developed for assessing all dimensions of psychopathy in childhood and adolescence.

A Youth Version (PCL:YV) of the PCL-R was developed by Forth, Kosson, and Hare [13] as a comprehensive assessment process to detect psychopathic traits in youths. Although detailed and exhaustive, this semi-structured interview is time-consuming since it requires 60 to 90 minutes to complete. Therefore, it is not easily accommodated in large-scale community studies. Moreover, Although its psychometric properties are generally strong [14,15], it does not allow for the direct opinion of the child being evaluated or parent report [15].

Among the most previously widely used clinical tools for psychopathic traits, the Antisocial Process Screening Device (APSD; [16]) is a 20-item parent-, teacher- or self-report rating scale based on the PCL-R. However, the alignment of its factor labels with the highest loading items on the corresponding subscale and their pathognomonic significance have been questioned [14,15,16,17]. Moreover, some concerns about its factors structure have been raised, with some studies finding a two-factor structure [18] and other studies finding a three-factor structure [19]. In addition, some authors have highlighted that the APSD clearly assesses negative behaviors, and its use as a self-report measure could lead to biased answers [20]; finally, some studies have found issues pertaining to its poor internal consistency, especially for the APSD’s CU subscale [21].

With its more traditional factor structure with ten subscales loading onto the three main dimensions (Interpersonal, Affective, and Lifestyle) of psychopathy, the Youth Psychopathic Traits Inventory (YPI) [20] is a 50-item self-report questionnaire designed to assess psychopathic traits in community samples of adolescents. A Short Form (YPI-S; [22]) has been validated in children, with 18 items equally distributed across three subscales, thus being easier to be administered in clinical settings. Nonetheless, this scale does not consider the antisocial dimension of child psychopathy, including poor behavioral control and proactive aggression, which would likely represent the most maladaptive feature of the condition [12]. In addition, it does not include a scale for Conduct disorder (CD) symptoms which could be important to study with psychopathic traits. Finally, not all studies reported optimal internal consistency for the YPI [23]. Internal consistency issues have also been reported for the Childhood Psychopathy Scale (CPS) [21] and the most broadly used Child Problematic Traits Inventory (CPTI) [24], although generally the psychometric properties for the CPIT are good. Perhaps the broader issue is that the scales lack a CD scale for those interested in the assessment of psychopathy in relation to CD.

In order to overcome the abovementioned limitations of the previous measures, Salekin and Hare [11] developed the Proposed Specifiers for Conduct Disorder (PSCD). The original version of the PSCD is a theory-driven measure composed of 24-items. It was designed to assess all the components of youths’ psychopathy, including grandiose-manipulative (GM) traits, callous-unemotional (CU) traits, daring-impulsive (DI) traits, plus CD/Oppositional Defiant Disorder (ODD) symptoms. A parent-report and a self-report version of the PSCD were then developed.

The first validation study of the PSCD was conducted by López-Romero and colleagues in 2019 [25]. The authors validated the parent-report version of the PSCD in a sample of 2,229 Spanish preschoolers aged 3 to 6 years. This was the first published study aimed to assess the psychometric properties of the PSCD. The version was adapted to be rated by parents and to include only age-appropriate items for preschoolers. The authors validated a four-factor model of the PSCD, including GM traits, CU traits, DI traits, and conduct problems. Associations with external criteria supported the validity of the PSCD, including measures of child fearlessness, conduct problems, reactive and proactive aggressive behaviors, and emotional problems.

Recently, Luo and colleagues [26] assessed the psychometric and structural properties of the self-rated PSCD in a sample of 1,683 Chinese adolescents aged 11 to 17 years. A Confirmatory Factor Analysis (CFA) supported the four-factor bifactor model, showing good internal consistency. This model showed generally high convergent associations with alternative measures of psychopathy, namely, the APSD, the YPI, and the Inventory of Callous Unemotional traits (ICU) [27].

### The Current Study

The PSCD construct validity has been established by a previous study by Luo et al. [26], as well as its associations with other measures of maladjustment. However, Luo et al. [26] examined the PSCD relation to the external criteria using only self-report measures. Some studies examined parent and child reports together and pointed out that parent measures had a greater magnitude of effect in predicting some outcomes [17,28]. Moreover, as the manifestations of the psychopathy dimensions are pervasive and not limited to a single context, teachers’ reports of children’s behavior at school could provide precious information. It is important to evaluate the relationships between the PSCD dimensions and low levels of youths’ prosocial behaviors, often indicated as a risk sign for future negative outcomes [29,30]. Moreover, although some measures assessing youths’ psychopathy are available in Italian, none of them have been validated. To sum up, the current study aimed to further validate the PSCD, testing the associations between the self-reported PSCD scores and parent- and teacher-reports of emotional/behavioral difficulties and prosocial behaviors. Given the similarities between Luo and colleagues’ study and our sample, we hypothesized to find similar results regarding the PSCD factor structure and its internal consistency. We also hypothesized that the PSCD scores would be associated with higher Conduct Problems and Hyperactivity Behavior and lower Prosocial Behavior [12].

## 2. Materials and Methods

### 2.1. Subjects

We enrolled 536 Italian students (47.76% male) aged between 11–14 years (males mean age = 12.20, SD = 0.65; females mean age = 12.60, SD = 0.60), consisting of 87 (16.20%) 6th graders, 360 (67.20%) 7th graders and 89 (16.60%) 8th graders, among which 88% of participants were Caucasian (12% from Africa). Of the whole sample, 492 students agreed to participate in the study. Thirty-four language teachers and the students’ caregivers were also involved. Regarding caregivers, most of the informants were mothers.

### 2.2. Procedures

Before completing any questionnaire, all the teachers, parents, and youths who agreed to participate in the study signed a written informed consent. Research assistants informed the participants that their participation in the study was voluntary, and they could withdraw from the study at any time. In addition, participants were assured of the confidentiality and anonymity of their answers. Throughout the study, research assistants were available to answer participants’ questions. While sitting in a quiet classroom, students were asked to complete the PSCD, which required approximately 15 min. Teachers completed the SDQ for each student. The SDQ was provided by a researcher who returned the completed SDQs in approximately one week. During a school meeting, parents also completed the SDQ. The study was conducted according to the guidelines of the Declaration of Helsinki and approved by the Institutional Review Board of Istituto Comprensivo Milani (PTOF, prot. 456 2019) and by the Institutional Review Board of Istituto Comprensivo G. Sani (PTOF, prot. 8 2019).

### 2.3. Measures

Students completed the PSCD [11]. The PSCD includes four domains, namely: Grandiose-Manipulative—an interpersonal component, intended to measure grandiosity, superficial charm, manipulation, and deceitfulness (thus labeled “Grandiose–Manipulative” or GM traits); Callous-unemotional—an Affective dimension, assessing callousness, uncaring affect, and disregard to others (thus labeled “Callous–Unemotional” or CU traits); Daring-Impulsive – similar to a Lifestyle domain, including daringness, sensation seeking, recklessness and irresponsibility (thus “Daring–Impulsive” or DI traits); and Conduct Disorder—the antisocial component (also referred to as CD traits), designed to measure the four main categories of CD symptoms (aggression to people and animals, destruction of property, deceitfulness or theft and serious violation of rules). A four-factor structure was initially theoretically proposed by Salekin and Hare in 2016 [11] for the self-rated PSCD, and its 24 items were equally distributed across the four subscales, each including six items. Items are rated on a three-point Likert scale, with responses ranging from 0 (not true) to 2 (true); a Total Score includes all 24 items. A bilingual researcher (forward) translated the PSCD from English to Italian. Then, a different bilingual researcher back-translated the questionnaire from Italian to English. The final version was further discussed by the above-mentioned translators and one of the original authors of this measure (R. T. Salekin) until they reached an agreement. No cultural adaptations were needed.

We asked teachers and parents to complete the Italian version of the SDQ [31]. The SDQ includes 25 items, and answers are provided on a 3-point Likert scale. The SDQ includes five subscales assessing Conduct Problems (e.g., bullying); Hyperactivity (e.g., squirming); Emotional Problems (e.g., worrying), Peer Problems (e.g., disliked by other children), and Prosocial Behaviors (e.g., helping). When all the items are filled in, the subscales’ scores range from 0 to 10. Higher scores indicate more behavioral problems, except for the prosocial behavior scores. In this study’s sample, the SDQ reliability was generally satisfactory, as demonstrated by the mean internal consistency of subscales (Cronbach’s α): 0.73 for Conduct Problems, 0.77 for Hyperactivity, 0.81 for Emotional Symptoms, 0.80 for Peer Problems, and 0.82 for Prosocial Behaviors.

### 2.4. Statistical Analysis

As a preliminary analysis, we performed descriptive statistics for all scales with SPSS 23.0. Analyses were conducted with 466 students with complete data (missing data, *N* = 26 about 5% of the sample, were excluded from the analyses). We calculated means and standard deviations for the PSCD total score, the PSCD subscales, and each item. A series of confirmatory factor analyses (CFAs) were conducted to test the best fitting model using MPLUS 7 and MLR as estimators with robust standard errors. The competing models of the PSCD structure were: Model 1: a one-factor model (all the 24 items loaded into a mono-factorial structure); Model 2: a four-factor model following the original definition as proposed by Salekin [12] (the 24 items of the PSCD as observed variables and the four factors as latent and correlated constructs-GM, CU, DI, and CD -, with each item specified to load on only one factor); Model 3: a second-order factor model with the four-factors as first-order constructs (see the previous model) and a second-order factor representing a general PSCD psychopathy syndrome; Model 4: a four factors bi-factor model where a general factor measured all the items directly and, at the same time, items loaded into their respective specific factors (GM, CU, DI, and CD). CFA fit indices included the comparative fit index (CFI) and root mean square error of approximation (RMSEA). Results were interpreted as follows: CFI values above 0.95, and RMSEA scores below 0.05, were considered indices of a good fit, whereas CFI larger than 0.90 and RMSEA smaller than 0.08 were considered indices of adequate model fit [32]. The comparison between competing factorial models has been analyzed using the AIC and the BIC values, and the ∆CFI.

We then evaluated the internal consistency of the PSCD score using the mean interitem correlations (MIC) and the ω_H_ for the general factor [33,34]. This index is usually used in short item scale, and it is used in Luo et al. [26]. The ω_H_ represents the ratio of the variance of the general factor compared to the total test variance, which reflects the percentage of systematic variance in unit-weighted total scores that can be attributed to the individual differences on the general factor. When ω_H_ values are high (≥0.80), relative to the percentage of variance explained by the group factors, it means that the total raw score could be interpreted as reflecting the target construct [35]. Moreover, the ideal range of MIC is 0.15 to 0.50; less than this, and the items are not well correlated and not measuring the same construct or idea very well. More than 0.50, and the items are close as to be almost repetitive. Multiple-Group analyses across gender were also conducted. Finally, we examined the correlations among the PSCD Subscales and Total Score, and Strengths and Difficulties Questionnaire Parent and Teacher report scores and independent samples t-tests were used to explore gender differences in the PSCD scores.

## 3. Results

### 3.1. Descriptive Statistics

Means and standard deviations of the 24 items of the original version of the scale are shown in Table 1.

### 3.2. Psychometric Properties

Table 2 shows the fit indices of the competing models. The four models tested showed poor fit indices, although the best fitting model was the four factors bi-factor structure (Model 4). Besides, the comparison between models 1–4 considering AIC and BIC values and the ∆CFI (always > 0.02) suggested Model 4 as the best fitting, and thus it was accepted as the final model. Looking at the solution, we decided to delete the items that were non-significant in the general factor (items: 3, 12, 14,16, 17) (see Model 4 revised). From GM traits, this included item 3. From CU traits, this included item 12; from DI traits, this included items 14, 16, and 17. Following the Modification Indices (MI = 12.927) we decided to set the loading of item 5 into the CU instead of the GM as in the original solution [12]. The final fit indices of this modified model showed acceptable fit indices.

**Figure 1:**
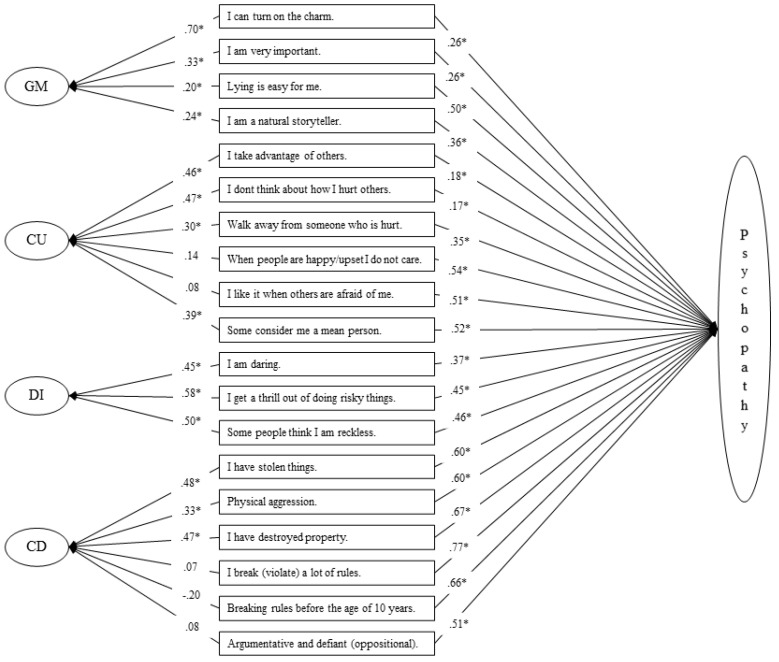
Factor loadings of the final four-factors Bifactor Model (standardized estimates). Abbreviations: GM, Grandiose–Manipulative; CU, Callous–Unemotional; DI, Daring–Impulsive; CD, Conduct Disorder. * *p* < 0.001.

Figure 1 shows the factor loadings. This final model showed some similarities with the model found by Luo and colleagues [26]: in particular, item 17 resulted as not significant also in this Bifactor model. The general factor reflects quite well on the indicators of all the specific items. However, in line with other studies using bifactor models [36], unexpected results were found for five factor loadings measuring specific factors. The loadings of the items 10-11 (CU Factor), and 22-23-24 (CD Factor) resulted in not being significant in measuring these specific factors, although these items were theoretically assumed to be indicators of these factors. Overall, however, the findings demonstrate that covariance among observed indicators can be well accounted for by a latent general factor, reflecting common variance among all indicators, and four latent group factors, reflecting additional common variance for subsets of indicators.

As also made by Luo and colleagues [26], we compared the 24-item scale with the reduced 19-item version regarding their ability to identify youths with high psychopathy features (PSCD mean total score > 1). In our sample, the 24-item PSCD identified 46 youths (8.6%) with high psychopathy features, while the 19-item scale identified 42 youths (7.8%). The difference in proportion was significant (χ^2^(1) = 412.54, *p* < 0.001) and in favor of the original 24-item scale. However, it is important to mention that there was only a slight difference in the number of subjects identified and that the two versions of the PSCD are highly correlated (*r* = 0.96, *p* < 0.001).

Regarding internal consistency, the 19-item PSCD total score (items 3-12-14-16-17 eliminated and item 5 loaded into the CU factor) had a MIC value of 0.32. The subscales MIC values were all acceptable: 0.27 for GM, 0.26 for CU, 0.35 for DI, and 0.31 for CD. Moreover, the ω_H_ for the general factor was 0.80. We also tested for (in)variance across gender for the 19 items version of the PSCD. Configural and metric/scalar invariance were examined in sequence for gender groups. The model fit indices were acceptable for configural invariance (although the CFI was slightly lower than 0.90) (χ^2^ (276) = 468.082, *p* < 0.001; CFI = 0.898; RMSEA = 0.057 (0.048–0.066), metric and scalar invariance model (χ^2^ (313) = 461.817, *p* < 0.001; CFI = 0.905; RMSEA = 0.047 (0.038–0.056)). Besides, the difference test between configural and metric invariance models (χ^2^ (15) = 15.541; *p* = 0.413) and the difference test between metric and scalar invariance models (χ^2^ (37) = 11.885, *p* = 1.00) resulted non-significant, meaning that our final model of the PSCD was invariant across gender groups. Finally, Table 3 shows the descriptive statistics of the PSCD scores, with gender comparisons.

### 3.3. Correlations with SDQ Scores

Table 4 shows the correlation between the PSCD scores and the SDQ parent- and teacher-report scores. The PSCD total score was strongly associated with parent- and teacher-rated conduct problems and hyperactivity symptoms; it was also negatively associated with parent- and teacher-reported prosocial behavior (moderate correlations). Scales of PSCD showed moderate or strong positive associations with conduct problems and hyperactivity symptoms. Among PSCD dimensions, only CU scores were associated with parent- and teacher-rated peer problems; only CU and CD scores were negatively associated with prosocial behaviors in the home and school contexts (moderate correlation indices).

## 4. Discussion

As expected, and consistent with Luo et al. [26], findings from our sample of Italian young adolescents indicated that the self-report PSCD questionnaire supported an overall bi-factor model consisting of four factors (GM, CU, DI, and CD) and a general factor. It is important to note that recent evidence indicated that the bi-factor model presents some advantages compared to the higher-order model for describing the general psychopathy traits because it directly teases apart the unique contributions to the indicators of the general and specific factors [37].

Some modifications were necessary for the Italian version of the PSCD. Modification indices suggested that item 5 best loaded onto the CU factor instead of the GM, as in the original version of the PSCD. The Italian version of the PSCD does not include item 3 (“I am very good at most things”) and item 12 (“I rarely feel guilt or remorse”) of the original version of the PSCD. The item 3 mean score was generally high, suggesting that most of the subjects in our sample may have positively interpreted the statement (e.g., being competent and skillful). Item 12 assesses an emotional feature of CU traits, namely lack of remorse and guilt. Social desirability or dissimulation may have influenced the mean score of this item. Items 3 and 12 were also removed from the Chinese version of the PSCD, suggesting these items might not clearly represent the psychopathy dimensions in youths, or at least not in this sample. We also deleted items 14 (“I like a lot of change or adventure”), 16 (“I feel like I need a lot of stimulation”), and 17 (“I like to live in the moment”). It is unclear why these items did not load, and will need additional testing as with the PSCD. It is interesting to note that Luo et al. [26] maintained items 14 and 16 in the DI subscale. Waiting for new research that might shed novel light on the item distribution for the PSCD, we can also hypothesize that translation issues and/or cultural differences may explain the modifications needed for the Italian version of the PSCD. The PSCD total and factors internal consistency was acceptable, especially for MIC indices. Moreover, we did not find gender differences regarding the PSCD structure in this sample.

Our results showed that, based on the 19-item version of the PSCD, 7.8% of the students reported high psychopathy features. There are currently no data on the prevalence of psychopathy in Italian samples of early adolescents, and our study could provide some helpful information on this matter. Compared to Luo and colleagues [26], we found a higher rate of youths with high psychopathy features (7.8% vs. 5.8% for the refined versions; 8.6% vs. 5.2% for the 24-item versions). Cultural differences might explain this discrepancy in the prevalence of high psychopathy features. Findings showed that boys reported higher scores in all the PSCD scales and total scores than girls. Sex differences in levels of psychopathic traits have indeed been confirmed by previous studies using different measures. Frick, Bodin, and Barry [19] found that boys had significantly higher scores than females regarding Narcissism, Impulsivity, and CU traits assessed with the APSD. Similarly, Andershed, Hodgins, and Tengström [38] found that males reported higher scores in the Interpersonal, Affective, Behavioral, and Antisocial factors of the PCL:YV as well as higher CU traits assessed with the YPI (see also [39,40]).

Besides, this is the first study to explore the correlations between the self-report PSCD scores and the parent and teacher reports of emotional/behavioral difficulties and prosocial behaviors. The previous validation of the self-report PSCD [26] relied solely on self-report measures, which the authors themselves recognized as a major limitation of their study. As hypothesized, the correlations between PSCD scores and these external criterion variables supported associations between high levels of PSCD scores and high levels of behavioral problems, with some differences in the association between the PSCD single scales and the SDQ scores. The PSCD total score showed moderate and good positive associations with conceptually and clinically important variables, including conduct problems and hyperactivity behaviors in home and school contexts. Furthermore, the total score of the PSCD seemed to be also associated with low prosocial behaviors. 

Among the PSCD dimensions it is particularly interesting to note that those individuals with elevated GM traits appeared to be rated by parents and teachers as not having difficulty with peers (SDQ-Peer Problems). That is, children with elevated GM traits generally seemed to be able to get along with their peers at least in the eyes of parents and teachers, or at least not be detected by parents and teachers, as having problems with their peers even though they engaged in antisocial behavior (conduct problems). Similarly, those youth with elevated DI traits were also able to get along with peers despite engaging in antisocial behavior although interestingly teachers for both GM and DI youth noted their lack of engagement in prosocial behavior indicating that they may not have completely evaded detection. Only the CU dimension appeared to be associated with noted Peer Problems and low Prosocial Behaviors. Different explanations can account for these unique associations, which deserve further investigation. Youth with GM traits are thought to be able to evade trouble more easily. Similarly, youth with elevated Di traits are not thought to suffer from the same cognitive problems as those with CU traits. Empathy impairments may be more core to high CU traits [41,42,43]. CU youths’ inability to connect with other people’ feelings and needs may result in general carelessness toward others, making youths with CU traits less prone to prosocial actions and with shorter, less stable [44], and satisfactory friendships [45]. 

Findings need to be interpreted considering some limitations. First, the sample size was relatively small. The final solution from the bi-factor model showed that the general factor reflects quite well indicators of all the specific dimensions; however, not significant factor loadings emerged for two items of the CU dimension and three items for the CD dimension. Although this pattern needs to be replicated, the finding suggested that caution needs to be considered when interpreting CU and CD scores. Moreover, even though beyond the primary aims of this study, we did not include other measures to assess psychopathic traits. The external criteria of the PSCD are limited to emotional and behavioral difficulties; other criteria relevant to the psychopathy construct should be considered in future studies, such as cognitive functioning [46], emotional intelligence and empathy [42,47], and proactive aggression [48]. We did not have temporal stability data on psychopathic traits for this group, although there are considerable data to show modest to high stability for children and adolescents [6,49].

## 5. Conclusions

Despite these limitations, we explored the associations between the PSCD scores and a wide range of behavioral and emotional difficulties. Behavioral problems are widely prevalent disorders in youths, and since their frequency is increasing, more reliable measures are constantly required. Self-report measures are particularly needed for an accurate assessment of early adolescents and older youths [50,51], since as their autonomy increases, parents are no longer completely aware of all the aspects of their child’s life. The PSCD could help clinicians to identify youths who are at greatest risk and for whom targeted early interventions could be provided at a relatively low cost. The PSCD could also be an appealing instrument for different professional figures (e.g., social workers, pediatricians, psychologists): it has few items and could be easily added to screening batteries (e.g., large-scale epidemiological studies, evaluations in the school context). Moreover, the CD scale allows for assessing the four CD symptoms, as delineated in the DSM-5. However, further validation studies are needed before introducing the PSCD as a tool in clinical assessment procedures (e.g., test-retest reliability).

## Figures and Tables

**Table 1:** Means and standard deviations of the PSCD items.

Items	Min	Max	Skewness	Kurtosis	Mean	SD
1: I can turn on the charm in any situation	0	2	0.96	−0.12	0.47	0.62
2: I am a very important person	0	2	0.66	−0.62	0.60	0.66
3: I am very good at most things I do	0	2	−0.11	−0.44	1.21	0.59
4: Lying is easy for me	0	2	0.49	−0.95	0.71	0.72
5: I take advantage of others	0	2	2.20	4.19	0.21	0.47
6: I am a natural storyteller	0	2	0.42	−1.05	0.76	0.73
7: I don’t waste time thinking about how I may have hurt others	0	2	1.11	0.07	0.46	0.65
8: I can turn and walk away from someone who is hurt	0	2	1.53	1.13	0.36	0.63
9: When people are happy or upset I do not seem to care	0	2	1.73	2.16	0.26	0.49
10: I like it when others are afraid of me	0	2	1.42	0.95	0.38	0.72
11: Some people consider me to be a mean person	0	2	1.05	−0.23	0.50	0.69
12: I rarely feel guilt or remorse	0	2	0.25	−1.11	0.85	0.73
13: I am daring	0	2	−0.04	−1.12	1.02	0.73
14: I like a lot of change or adventure	0	2	−0.45	−0.87	1.28	0.69
15: I get a thrill out of doing risky things	0	2	0.45	−1.20	0.76	0.77
16: I feel like I need a lot of stimulation	0	2	0.15	−1.25	0.91	0.76
17: I like to live in the moment	0	2	−0.62	−0.99	1.33	0.75
18: Some people think I am reckless	0	2	0.46	−1.23	0.75	0.78
19: I have stolen things	0	2	1.46	0.70	0.39	0.67
20: I have engaged in physical aggression against animals or people	0	2	1.02	−0.66	0.54	0.79
21: I have destroyed property	0	2	1.55	1.09	0.36	0.65
22: I break (violate) a lot of rules	0	2	1.06	−0.01	0.47	0.65
23: I started breaking rules before the age of 10 years	0	2	2.47	4.94	0.21	0.54
24: I can be argumentative and defiant (oppositional)	0	2	0.47	−1.01	0.74	0.75

Abbreviations: PSCD, Proposed Specifiers for Conduct Disorder; Min, minimum; Max, maximum; SD, standard deviation.

**Table 2:** Fit indices of the competing models.

	χ^2^	Df	*p*	CFI	RMSEA	AIC	BIC
Model 1: Mono-dimensional	732.540	252	<0.001	0.758	0.064 (0.059–0.069)	21,679.174	21,977.556
Model 2: Four Factors (original as Salekin, 2017)	606.143	246	<0.001	0.818	0.056 (0.050–0.062)	21,532.517	21,855.763
Model 3: Second Order	621.857	248	<0.001	0.812	0.057 (0.051–0.062)	21,543.189	21,858.147
Model 4: four factors bi-factor	495.433	228	<0.001	0.865	0.050 (0.044–0.056)	21,451.710	21,849.552
Model 4 revised: four factors bi-factor *	344.469	133	<0.001	0.910	0.059 (0.051–0.067)	16,556.136	16,871.094

* The items: 3-12-14-16-17 were deleted, and item 5 was loaded into the CU factor.

**Table 3:** Descriptive statistics of the PSCD subscales and total score, with gender comparisons.

	Range		Total Sample (*N* = 536)	Males (*N* = 256)	Females (*N* = 280)			
	Min	Max	Mean (SD)	Mean (SD)	Mean (SD)	*t*	*p*	*d*
PSCD Total	0	31	10.01 (5.81)	11.35 (6.13)	8.68 (5.33)	−4.03	<0.001	0.46
GM	0	12	2.52 (1.68)	2.65 (1.66)	2.28 (1.67)	−4.66	<0.001	0.22
CU	0	6	2.18 (1.95)	2.60 (2.05)	1.88 (1.83)	−2.27	0.023	0.36
DI	0	11	2.54 (1.72)	2.80 (1.71)	2.20 (1.71)	−3.72	<0.001	0.35
CD	0	8	2.71 (2.64)	3.26 (2.80)	2.25 (2.37)	−3.57	<0.001	0.39

Abbreviations: PSCD Total, Proposed Specifiers for Conduct Disorder Total Score; GM, Grandiose–Manipulative; CU, Callous–Unemotional; DI, Daring–Impulsive; CD, Conduct Disorder.

**Table 4:** Bivariate correlations between the scores of the revised version of PSCD (19 items) and Strengths and Difficulties Questionnaire Parent and Teacher report scores.

	PSCD Total	GM	CU	DI	CD
PSCD Total	1				
GM	0.65 **	1			
CU	0.68 **	0.21 **	1		
DI	0.66 **	0.41 **	0.20 **	1	
CD	0.84 **	0.34 **	0.48 **	0.36 **	1
SDQ_P_EP	0.03	0.02	0.07	0.01	0.11
SDQ_P_CP	0.35 **	0.22 **	0.26 **	0.17 **	0.31 **
SDQ_P_HY	0.37 **	0.20 **	0.28 **	0.24 **	0.31 **
SDQ_P_PP	0.16 **	−0.03	0.31 **	−0.02	0.14 **
SDQ_P_PB	−0.21 **	−0.02	−0.28 **	−0.01	−0.22 **
SDQ_T_EP	0.07	0.02	0.10	−0.03	0.10
SDQ_T_CP	0.41 **	0.28 **	0.36 **	0.26 **	0.35 **
SDQ_T_HY	0.45 **	0.28 **	0.37 **	0.36 **	0.36 **
SDQ_T_PP	0.09	0.01	0.26 **	−0.01	0.02
SDQ_T_PB	−0.25 **	−0.13 *	−0.24 **	−0.19 **	−0.20 **

Abbreviations: PSCD Total, Proposed Specifiers for Conduct Disorder Total Score; GM, Grandiose–Manipulative; CU, Callous–Unemotional; DI, Daring–Impulsive; CD, Conduct Disorder; SDQ_P_EP, SDQ parent-report Emotional Problems; SDQ_P_CP, SDQ parent-report Conduct Problems; SDQ_P_HY, SDQ parent-report Hyperactivity Symptoms; SDQ_P_PP, SDQ parent-report Peer Problems; SDQ_P_PB, SDQ parent-report Prosocial Behavior; SDQ_T_EP, SDQ teacher-report Emotional Problems; SDQ_T_CP, SDQ teacher-report Conduct Problems; SDQ_T_HY, SDQ teacher-report Hyperactivity Symptoms; SDQ_T_PP, SDQ teacher-report Peer Problems; SDQ_T_PB, SDQ teacher-report Prosocial Behavior. * *p* < 0.05 ** *p* < 0.01. Correlation coefficients ≤ 0.10 are weak; 0.20 to 0.29 are moderate; ≥0.30 are good (Hemphill, 2003).

## Data Availability

The data are available from the corresponding author upon reasonable request.
